# Comparative Analysis of the Physical, Tactical, Emotional, and Mood Characteristics of Under-13 Soccer Players by Performance Level

**DOI:** 10.3390/jfmk9040237

**Published:** 2024-11-15

**Authors:** Aura D. Montenegro Bonilla, Sergio D. Rodríguez Pachón, Víctor Hernández-Beltrán, José M. Gamonales, Markel Rico-González, José Pino-Ortega, Jorge Olivares-Arancibia, Rodrigo Yánez-Sepúlveda, José Francisco López-Gil, Boryi A. Becerra Patiño

**Affiliations:** 1 Faculty of Physical Education, National Pedagogical University, Valmaria Cl. 183 # 5199, Bogotá 480100, Colombia; admontenegrob@upn.edu.co (A.D.M.B.); sdrodriguezp@upn.edu.co (S.D.R.P.); 2 Training Optimization and Sports Performance Research Group (GOERD), Faculty of Sport Science, University of Extremadura, 10005 Cáceres, Spain; vhernandpw@alumnos.unex.es (V.H.-B.); martingamonales@unex.es (J.M.G.); 3 Facultad de Educación y Psicología, University of Extremadura, 06006 Badajoz, Spain; 4 Department of Didactics of Musical, Plastic, and Corporal Expression, University of the Basque Country (UPV-EHU), 20600 Eibar, Spain; markel.rico@ehu.eus; 5 Faculty of Sport Science, University of Murcia, 30100 Murcia, Spain; josepinoortega@um.es; 6 AFySE Group, Research in Physical Activity and School Health, School of Physical Education, Faculty of Education, Universidad de las Américas, Santiago 7500000, Chile; jolivares@udla.cl; 7 Faculty Education and Social Sciences, Universidad Andres Bello, Viña del Mar 2520000, Chile; rodrigo.yanez.s@unab.cl; 8 One Health Research Group, Universidad de Las Américas, Quito 170124, Ecuador; 9 Management and Pedagogy of Physical Activity and Sport (GPAFD), Faculty of Physical Education, National Pedagogical University, Valmaria Cl. 183 # 5199, Bogotá 480100, Colombia

**Keywords:** sports, performance, training, cognitive process, physical qualities

## Abstract

**Background and Objectives**: Soccer is a sport characterized by various unpredictable situations in which physical abilities are associated with athletic performance. There are several capabilities that young soccer players must develop to adapt to the needs of the competition. This study analyzes the physical characteristics, tactical knowledge, emotional intelligence, and mood states of youth soccer players at different competitive levels. **Materials and Methods**: The sample consisted of 36 male soccer players with an average age of 12.65 ± 0.48 years, weight of 44.92 ± 7.49 kg, and height of 157.2 ± 0.08 cm. A cross-sectional correlational study design was selected. Inferential analysis was conducted via the RV coefficient to assess relationships between groups. Two-sample tests (Student’s *t* test or the Mann–Whitney *U* test) were used to assess the distribution of the samples. Standardized mean differences (i.e., Cohen’s *d*) were calculated as effect sizes. **Results**: For the yo-yo intermittent endurance test level 1, the Premier category showed higher speed (*p* = 0.01, *d* = 0.40) and superior estimated VO_2max_ (*p* = 0.01, *d* = −0.91). The statistically significant variables included the hamstring strength exercise of the hamstrings for the angle of rupture (*p* = 0.04, *d* = −0.04, *d* = −0.72), the COD-Timer 5-0-5 for contact time—5-0-5 (ms) (*p* = 0.04, *d* = 0.69) and 10 m—5-0-5 (s) (*p* = 0.02, *d* = 0.79), tactical knowledge of in-game performance (*p* = 0.01, *d* = −1.19), support level (*p* = 0.01, *d* = −1.27), decision-making ability (*p* = 0.01, *d* = 0.59), melancholy (*p* = 0.01, *d* = 0.59), confusion (*p* = 0.01, *d* = 0.56), and emotional intelligence (*p* = 0.04, *d* = 0.77). The Premier category presented slightly higher averages than did category A. In the assessment of running-based anaerobic sprint test power (*p* < 0.05, *d* = 0.83) and mood states (*p* < 0.05, *d* = 0.59), players in category A presented higher results. **Conclusions**: The performance capacity of youth soccer players encompasses a multidimensional complexity that includes physical, tactical, emotional, and psychological aspects, which vary among players of the same age.

## 1. Introduction

Soccer is a sport distinguished by the multiple random situations that may take place, where physical capabilities manifest sports performance; these physical capabilities allow athletes to adapt to competition demands [1]. Many of these actions are manifested by the integration of strength [2], speed [3], resistance to explosive efforts [4], and power [5,6], which, in turn, allow the development of actions such as acceleration, deceleration, jumps, and turns, among others. Understanding the behavior of these physical variables is crucial for sports processes [7] because characterizing them in youth soccer according to players’ competitive level will help build more individualized planning processes [8].

However, owing to the unpredictable nature of soccer, it is difficult to isolate and determine which variables determine the specific performance of youth soccer players [9]. Chan et al. [10] determined differences in physical capabilities between youth and professional soccer players and concluded that professional players showed superior performance in terms of acceleration and deceleration, whereas youth soccer players displayed greater performance in terms of speed. However, some studies have reported that there are relationships between changes in direction ability and sprinting in young soccer players [11]. Similarly, another study determined positional differences in soccer players aged between 8 and 18 years and concluded that there is no difference among U9-U15 players in terms of physical characteristics, except for dribbling ability, and that predominant physical capacities of strength, power, and speed could favor the selection of players in some positions [12].

However, it is necessary to recognize that other performance indicators can optimize sports processes in the long term [13]. Sanchez et al. [14] sought to determine physical performance and technical/tactical behavior in young soccer players. Several studies have focused on establishing players’ performance on the basis of perceptual/cognitive variables among age groups and performance levels [15]. Other studies highlight tactical knowledge, which is determined by the athlete’s problem-solving ability and reflected at different tactical levels, such as teams, small groups, and individual actions [16]. Similarly, contextual variables influence tactical capacity, as competitive behavior responds to game situations, as do external conditions that influence athlete behavior in unstable and uncertain environments [17].

Similarly, tactical performance was studied via various instruments. One of the main instruments is the Game Performance Assessment Instrument (GPAI), which has been used by teenage soccer players to determine tactical knowledge through decision-making and motor efficiency [18]. In this sense, tactical performance in youth soccer depends on factors associated with game intelligence, observation ability, and problem solving [19,20]. Understanding these physical and tactical variables can help recognize player profiles, given the individual demands that each playing position allows [21,22,23]. However, other performance variables are associated with the influence of emotions and mood states (MSs) [24,25].

Conducting a study that allows comparisons of physical, tactical, emotional intelligence (EI), and mood variables in youth soccer players could help build profiles that provide information about the characteristics that determine players’ performance and, thereby, chart roadmaps that allow the selection of sports talent from a much broader perspective. In the same vein, other studies have considered EI as a variable that influences sports outcomes [26]. Thus, emotions play a major role in performance manifestation, as they favor perceptual, cognitive [27], motivational, and emotional processes to manifest motor expression through decisions [28]. A meta-analytical study sought to corroborate the relationship between emotions and sports performance and concluded that EI can serve as a possible predictor of sports performance [29]. Likewise, there are psychological factors associated with obtaining competitive results, among which motivation and MS stand out [30].

Variation in MS has been studied in various studies, recognizing that it is related to sports success or failure [31]. Therefore, it is necessary to characterize the MS of youth soccer players to design pedagogical strategies that favor sports performance. The scientific literature has provided essential insight into the human athlete (HA) as a complex, adaptive, and multifunctional system because various interconnected structures play a leading role in its development [32]. The balanced optimization of the dimensions is essential for the integral development of the HA.

In this regard, and following the approach proposed by Bellistri et al. [33], it is necessary to incorporate a high quantity and diversity of performance indicators that could serve as input to recognize the interactions that occur in athletes and, thus, understand what happens in sports with high cooperation/opposition, including physical, tactical, emotional, and psychological characteristics. No study has evaluated the physical capabilities and other performance variables associated with the tactical, emotional, and psychological components of youth soccer players. From this, relationships that characterize the profiles of the players according to their competitive level are established. This study analyzes the physical characteristics, tactical knowledge, emotional intelligence, and mood states of youth soccer players in the formation stage.

## 2. Materials and Methods

### 2.1. Design

This was a cross-sectional correlational study [34]. The sampling type is probabilistic from study groups. Under the described experimental design and taking a type I error of 5% and a nominal power of 80%, the sample size was determined via Cohen’s *d* statistic, with the detection of strong differences between groups (category A and Premier category) as a reference. The sample sizes under the given conditions were 17 and 19 athletes, respectively (power = 0.801).

### 2.2. Participants

The evaluated sample consisted of 36 male soccer players in the formation stage, with an average age of 12.65 ± 0.48 years, weight of 44.92 ± 7.49 kg, and height of 157 ± 0.08 cm. The participants were part of the 2010 Premier category (*n* = 19) and the 2010 category (*n* = 17) of the same soccer club in Bogotá, Colombia.

The Premier category is classified in the local tournament as the highest performance category, whereas the A category is one step below the Premier category. The inclusion criteria were (i) not presenting any injury, pathology, or surgery that would prevent participation in the study; (ii) signing assent and informed consent; (iii) being born in the year 2010; (iv) completing all tests; (v) training at least four times a week; and (vi) having a minimum of two years of experience playing soccer.

Each player provided assent, and their parents provided informed consent for voluntary involvement after receiving detailed explanations of the study’s objective, scope, and procedures. Each process was developed under the principles established by the Helsinki Declaration [35] and the 8430 resolutions of the Ministry of Health of Colombia [36], declaring the study as low risk according to Colombian regulations on the basis of noninvasive research standards and guidelines. Finally, the study received approval from the National Pedagogical University’s ethics committee (340ETIC-2024).

### 2.3. Procedure

There are different protocols for the assessment of physical capacity, tactical knowledge, EI, and MS. The evaluation is categorized into four phases: (i) evaluation of physical capacity; (ii) evaluation of tactical knowledge; (iii) evaluation of EI; and (iv) evaluation of MS. The evaluation of physical capacity was carried out over five days. Initially, on the first day, the following basic anthropometric measurements were taken: body mass and height. The study used the My Jump Lab (v.2, Spain) application for evaluating physical capacities to record the necessary information, which was based on extended leg length (cm), the length of the trochanter to the ground (cm), and the length of a squat with a 90° angle. The second day was dedicated to assessing squat jumps (SJs), countermovement jumps (CMJs) (*r* = 0.997, *p* < 0.001), and single-leg countermovement jumps (SLCMJs) (*r* = 0.995, *p* < 0.001). On the third day, speed tests were performed at 5, 10, and 15 m with the iPhone application Runmatic (*r* = 0.94–0.99, *p* < 0.001), as were changes in direction (COD-Timer 5-0-5) (*r* = 0.964; 95% confidence interval (CI) = 0.95–1.00; standard error of the estimate = 0.03 s; *p* < 0.001). Yo-yo intermittent recovery test level 1 (YYIE1) (ICCs between 0.69 and 0.97) was performed on the fourth day, and the running-based anaerobic sprint test (RAST) (ICC = 0.88) was performed on the fifth day. The second evaluation phase spanned four days for the assessment of tactical knowledge. The GPAI evaluation was performed on the basis of small-sided games in a space of 16 × 12 m in groups of three players. The 3 vs. 3 situations were recorded with goalkeepers in the same area as the preparatory game and with an equal number of players.

This was carried out in four sessions, each with a 1:1 activity ratio for recovery, as outlined in Table 1. Notably, each of the evaluations of the first two moments was separated by 48 h after the training session or previous competitive match. The IE evaluations were carried out on days 10 and 11, whereas the evaluation of the MS data was carried out on days 12 and 13. The Bar-On EQ-i: YV was used, specifically the 60-item version, considering inconsistency indices in each response EI. The test demonstrated reliability, with a Cronbach’s alpha coefficient for the total scale of 0.76, and the reliability indices for each factor ranged from 0.63–0.80.

In the Bar-On Emotional Quotient Inventory: Youth Version (Bar-On EQ-i: YV) and the TEAD-R applications, each of the four researchers were presented with any questions that arose. Finally, all the data were collected under standard environmental conditions with respect to time (4:00 to 6:00 p.m.) and temperature (between 15° and 22°) from April to August 2023. Each player was asked for 15 min before the start of training to carry out the standardized 8 min warm-up protocol, which consisted of joint movements and lateral and frontal displacements [37].

### 2.4. Instruments

Researchers used a body composition scale (Omron scale Hbf-514C, Kyoto, Japan) with a precision of 0.1 kg to determine weight and a portable stadiometer (Seca, 213). The method selected for strength evaluation through SJs and CMJs was the My Jump 2 video camera recording application. The variables evaluated in both jumps were jump height (cm), flight time (ms), average speed (m/s), force (N), and power (W). [38]. Changes in direction (CODs) were assessed via the iPhone application COD-Timer. The variables assessed were 5-0-5 (s), average speed—5-0-5 (km/h), contact time—5-0-5 (ms), 10 m—5-0-5 (s), and COD deficit (s) [39], and the Runmatic iPhone app [40] was used for running mechanics over 5, 10, 15, and 20 m. SLCMJ was used to assess asymmetries through intelligent application. The variables assessed were asymmetry contact time %, asymmetry time of flight %, contact time left (ms), contact time right (ms), time of flight left (ms), and time of flight right (ms) [41]. The hamstring strength test was used to evaluate the torque (Nm) and angle of rupture (°). The YYIE1 was used to assess intermittent endurance capacity, and the variables assessed were speed (km/h), distance (m), and estimated VO_2max_ (mL/min/kg). [42]. The running anaerobic sprint test (RAST) allows for the assessment of players’ anaerobic capacity through the following variables: time in each of the 6 sprints, power in each of the 6 sprints, minimum power, maximum power, and percentage of fatigue. The test involves six sprints over a 35 m distance, with a 10 s recovery between each sprint [43].

The GPAI [44] was used to assess the tactical knowledge of the athletes. For the assessment of tactical knowledge in both groups, the assessment was conducted by two independent assessors who were blinded to the category to which the player belonged to reduce bias. The players were from the same club, and the assessment was approved by the coaching staff. For this purpose, the players were randomly assigned according to the lists provided by the coaches in charge. Thus, developing evaluations with mixed players helped reduce bias. Players were assigned to groups of three, and for each group, four situations were evaluated (3 vs. 3 free play, 3 vs. 3 without goalkeepers and small goals, 3 vs. 3 with goalkeepers and small goals, 3 vs. 3 with goalkeepers and small goals and changes in direction). Assessments were conducted over four days for each group and were designated every 48 h. Each training session lasted 120 min and was recorded for later evaluation with the GPAI tool (Table 2).

The Bar-On EQ-i: YV questionnaire was used for the EI assessment. The 60-item version was used to account for response inconsistency [45]. The Mood Test for Performance Athletes (TEAD-R) was used for MS determination [46].

### 2.5. Data Analysis

Researchers have conducted a preliminary data evaluation, assessing consistency, coherence, and uniformity. Following this, the variables were reorganized and standardized into categories. The 67 variables are consolidated into 15 groups. Multiple factor and inertia analyses were subsequently used to examine the relationships between these groups. Correlations between groups were identified, and relationships between variables were analyzed in detail.

Additionally, contribution, representation quality, and dimensionality analyses were considered. Inferential analysis was carried out via the RV coefficient to assess relationships between groups. For specific variable details, two-way tests (Student’s *t* test or Mann–Whitney *U* test) were used after validating the assumption. Effect sizes (ESs) were determined via Cohen’s d test or the Wilcoxon test on a case-by-case basis. The following *p* values were established: * *p* < 0.05, ** *p* < 0.01, and *** *p* < 0.001. All analyses were performed via RStudio version 4.1.0 software (RStudio, INC, Boston, MA, USA, 2016).

## 3. Results

### 3.1. Analysis of Results by Category (Premier vs. Category A Comparison by Structures)

#### 3.1.1. SJ and CMJ

The CMJ test (*p* = 0.96) and SJ test (*p* = 0.79) did did not yield statistically significant differences. The CMJ test revealed that, on average, both categories had similar jump heights, flight times, strengths, speeds, and power values. Similarly, in the SJ test, the mean jump height, flight time, strength, speed, and power did did not differ significantly between category A and Premier.

#### 3.1.2. SLCMJ

With respect to contact time and flight time in terms of asymmetry, no significant differences were observed between the groups (*p* > 0.05). For contact time and flight time for the left and right sides, no significant differences were observed between groups in any of the respective variables (*p* > 0.05). This implies a similarity in contact and flight times between the left and right sides for both categories.

#### 3.1.3. YYIE1 and RAST

The data presented in Table 3 indicate that for the YYIE1 test, the Premier category had a higher average speed than did category A, with a statistically significant difference (*p* = 0.01). Additionally, in terms of distance covered, the Premier category recorded a higher average (*p* = 0.01, *d* = −0.917). Premier players presented higher average values of maximum oxygen consumption (estimated VO_2max_) (*p* = 0.01, *d* = −0.917). These results suggest that there appear to be no differences in the development of explosive efforts evaluated with the RAST when comparing both categories of the present study, since no differences were reported for minimum power, maximum power, and fatigue.

#### 3.1.4. Hamstring Strength

The data revealed that (Table 4), with respect to torque (Nm), Premier-category players had a slightly greater average than did those in category A. However, this difference was not statistically significant (*p* = 0.71). On the other hand, for the breaking angle (°), the Premier group presented a greater average (*p* = 0.041, *d* = −0.72), suggesting that the Premier group presented a greater breaking angle than did Group A.

#### 3.1.5. Speed and COD-Timer 5-0-5

Considering the speed test results (Table 4), no significant differences were identified between the categories in the 5 m (*p* = 0.98), 10 m (*p* = 0.11), 15 m (*p* = 0.22), and 20 m (*p* = 0.19) speed tests. The Premier players had a slightly lower average than Group A. Despite the difference in the average, no statistically significant differences were found between the categories. Considering the sports category, these data revealed better performance in the Premier-Category athletes because they needed less time to complete the distances.

With respect to the time recorded in the COD-Timer 5-0-5 test, both categories presented similar times. There were no significant differences between the groups (*p* = 0.70). Similarly, for the average speed during the 5-0-5 test, there was no statistically significant difference (*p* = 0.20). In terms of contact time, a significant difference was observed between the groups (*p* = 0.04, *d* = 0.69). This suggests that Premier players presented shorter contact times, indicating greater agility and responsiveness than other categories did. For the 10 m variable, a significant difference was found between the groups (*p* = 0.02, *d* = 0.79), with Premier players showing a slightly lower average time. With respect to the COD deficit, Premier players presented a slightly higher average value.

#### 3.1.6. Tactic Knowledge

The results consistently showed that Premier players present significantly higher levels of three of the analyzed variables. These significant differences were presented in in-game performance (*p* = 0.001, *d* = −1.19), support level (*p* = 0.001, *d* = −1.27), and decision-making (*p* = 0.001, *d* = 0.59) between Premier and Category A. However, there were no significant differences in skill execution between the two categories (Table 5).

#### 3.1.7. Emotional Intelligence and Mood States

Table 6 shows statistical comparisons between categories regarding various variables of EI and MS. In the case of the tension variable, Premier players presented a greater average than Group A players did, although the difference did did not reach statistical significance (*p* = 0.09). With respect to the melancholy variable, the results revealed a significant difference between categories, with a greater average for Premier players (*p* = 0.001, *d* = 0.592). In the case of the EI assessment, there were statistically significant differences in favor of the Premier category players (*p* = 0.04, *d* = 0.771). These findings indicate notable differences in these structures between categories, suggesting possible implications for emotional management and the overall well-being of players in both groups. With respect to vigor (*p* = 0.02, *d* = 0.386) and confusion (*p* = 0.001, *d* = 0.567), Premier players presented a significantly greater average than Group A players did.

### 3.2. Multiple Factor Analysis

The values of the RV coefficient (Table 7) identified relationships between sets of variables. Thus, when comparing between groups, there were strong interactions between the CMJ and SJ tests (RV = 0.83; *p* = 0.001) and between speed and the RAST (RV = 0.48; *p* = 0.001); another evident relationship was between the SJ and RAST (RV = 0.47; *p* = 0.001). These values reflect highly significant relationships.

**Figure 1:**
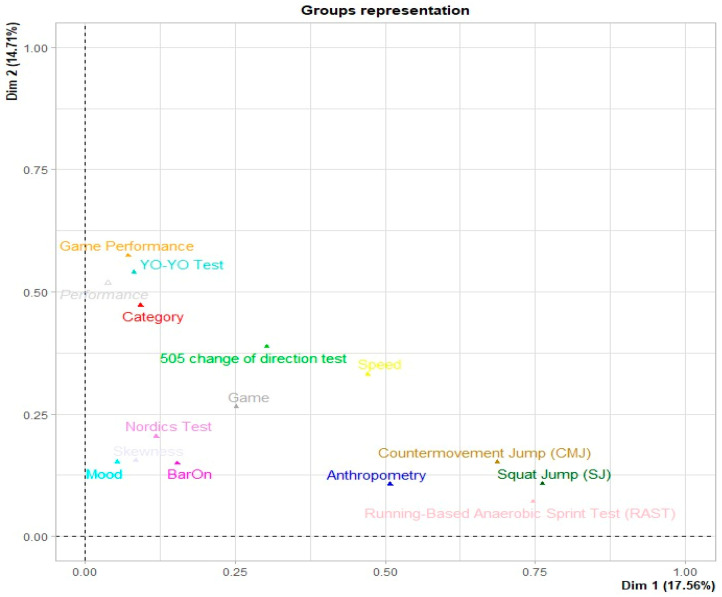
First view of the study variables.

**Figure 2:**
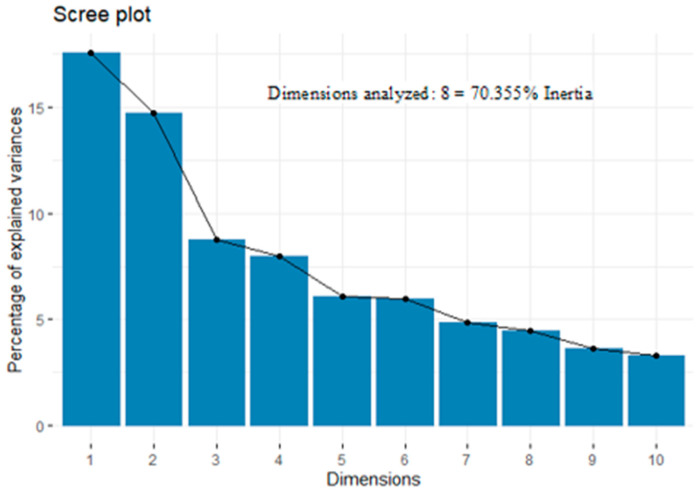
Eigenvalue analysis by dimension.

Regarding the plane presented in Figure 1 for the variable groups, the arrangement of the groups can be observed on the basis of the interaction they presented. Variable groups such as the RAST, SJ, and CMJ variables are related, whereas other variables are related to game performance, category, and direction changes. In contrast, the variables SJ, CMJ, and RAST had little relationship with the variables game performance, YYIE1, and category. Finally, the first two dimensions contribute 32.27% of the total information. In multiple factor analysis, we can observe variables from different dimensions, allowing us to study them from various perspectives. To demonstrate how much information was recovered per dimension, an eigenvalue analysis was conducted (Figure 2), which revealed that 70.35% of the information was recovered. Additionally, these data were distributed across eight dimensions.

### 3.3. Results of the Relationships Among the Variables Under Study in Youth Players of Different Competitive Levels (Premier and Category A) on the Basis of Multiple Factorial Analyses

An analysis of physical variables, tactical knowledge, EI, and MS among youth soccer players of different competitive levels revealed that there were multiple identified profiles. Thus, it is possible to determine the eight profiles outlined below (Table 8).

## 4. Discussion

This study analyzed the physical characteristics, tactical knowledge, emotional intelligence, and mood states of youth soccer players in the formation stage. Soccer is a sport that encompasses multiple abilities; conditional variables (strength, power, speed, and CODs) play a leading role. However, it is necessary to acknowledge that other qualities, such as tactical and emotional qualities, influence players’ sports training and education. Analyzing the influence of different emotional, psychological and tactical variables on players’ performance will allow us to predict certain behaviors and detect moments of low motivation toward training. In this way, generating player profiles according to specific variables will allow coaches to determine and characterize training sessions to encourage and produce high levels of attention and motivation in athletes. This is important because it allows us to understand the sports training of young footballers from a much broader perspective.

Thus, this study considers a variety of emotions, revealing some emotional characteristics where players competing at higher performance levels exhibit more negative moods. Although studies evaluating physical characteristics, tactical components, EI, and MS together have not been reported thus far, studies have associated emotional components with performance [27,28], such as the findings in this study, as players in the Premier category exhibit lower MS than those in category A. In this vein, Gershgoren et al. [26] affirm that anxiety is the emotion most studied in the sports world, but other emotions need attention.

The fifth profile of this study revealed that players with high levels of melancholy were associated with high levels of hostility, confusion, and tension. Additionally, these athletes correlated with elevated contact times in the right leg in the asymmetry test, indicating that melancholy levels do not affect physical performance (except for this finding in one test, which could very well be incidental due to the small sample size). Furthermore, research by Lane et al. [47] demonstrated that optimal performance was associated with higher scores for vigor, calmness, and happiness, along with lower scores for scales of anger, confusion, depression, fatigue, and tension.

In this context, Rumbold et al. [24] reported that collective emotions were strongly linked to the group-based emotions of an individual, which can be related to the findings of this study, where different categories showed emotional characteristics that can specifically speak about the athletes, with Premier players exhibiting significantly greater average MS than Group A players.

The study developed by Kopp and Jekauc [29] revealed a correlation between EI and sports performance, demonstrating a small but significant association (r = 0.16), which could be associated with the results of the multiple factorial analysis, where EI and tactical aspects presented a moderate–low correlation (RV = 0.14). Consistent with the above, the fourth profiling of the multiple factorial analysis in this work reveals that players with optimal levels of conditional hamstring strength and asymmetry are associated with high levels in the EI test, BarOn. EI can influence aspects of sports performance, since possessing good emotional management skills benefits competition-related situations, which is reflected more in athletes demonstrating better performance [48,49]. With respect to other types of variables, weak associations were observed between tactical performance indices and somatic maturity, functional capacity, and anthropometric attributes (r < 0.40) [50]. These qualities have a limited impact on tactical performance, similar to what was found in this research, where players with better levels of game intelligence and tactical performance did not necessarily exhibit the best physical variables.

Players who are exposed to longer training times and more training sessions generally show good performance in terms of in-game intelligence traits, similar to what is presented in this study, where players in the Premier category exhibited better results in three out of four tactical variables, highlighting that this group trains more days than does category A [15]. Considering that soccer involves numerous randomly occurring situations, it is important to note that players are constantly experiencing perceptual/cognitive processes. In this context, quicker and optimal decision-making allows for greater cognitive efficiency [51]. This can be associated with the present study, as players with higher tactical intelligence scores are better at decision-making, skill execution, and support.

With respect to physical variables, certain relationships were discovered that can be contrasted, for example, with the work developed by Comfort et al. [52], which found good associations of relative strength with 20-m sprint performance (r = −0.672, *p* < 0.001), similar to what was evidenced in this research, where strength performance moderately interacts with speed results (RV = 0.33); this consistency supports the idea that strength plays a relevant role in optimizing performance in short sprints.

In this vein, the study conducted by Carnevale et al. [53] demonstrated that resistance training with and without an external load was effective, as it improved jumping and aerobic endurance. The CMJ results revealed significant improvements in CMJ performance in both groups [SG: *p* < 0.001; OG: *p* < 0.001], most effectively in the OG (*p* < 0.004). These findings could be linked to one of the profiles given in this study, in which athletes with a high level of jumping power (SJ) also correlate with strength and exhibit high levels of different powers in the RAST. This connection between jump performance and anaerobic endurance highlights the importance of addressing multiple components of physical performance in an integrated manner, which could be explained by the ability to generate force. Therefore, specific training to improve strength in soccer is recommended.

Recent studies in young female soccer (U12) utilizing the RAST revealed that an 8-week intervention on anaerobic performance led to significant improvements, favoring the evaluation of different competitive levels [54]. Similar results were found in this study, where statistically significant differences in time and power values in response to players’ competitive level were established when anaerobic performance was evaluated via the RAST. Therefore, anaerobic and aerobic systems must be used to correctly perform sprints and races at moderate intensities, which are required by both parts of the competition in soccer. Additionally, strength training is recommended to reduce the number of sports injuries.

In other studies, young soccer players revealed physical characteristics and techniques in response to playing position [55]. Another study reported that defenders are faster than goalkeepers and midfielders and that defenders and midfielders are the positions with higher VO_2max_ levels [56]. This case is similar to the findings of the present study, where there are profiles associated with position, highlighting that they are differentiated not only by physical variables but also by tactical aspects, moods, and EI.

This study addressed various performance qualities of young soccer players, highlighting the relationships among physical, tactical, and emotional aspects. The results underscore the importance of managing emotions, developing tactical intelligence, and recognizing the relevance of physical abilities in the soccer context. The associations between strength and speed, as well as improvements in jumping and endurance with training, suggest that a holistic approach to training is essential for the comprehensive development of young players. These findings can inform more effective training strategies and contribute to the overall understanding of performance in youth soccer. Therefore, comprehensive training must be carried out in football to provide players with great abilities to develop them during the competition.

The results underscore the importance of considering not only conditional and tactical structures but also the psychological and emotional aspects of players. Although players at higher competitive levels may demonstrate superior tactical and EI characteristics, high levels of psychological pressure derived from competition can also lead to the expression of high levels of variables such as tension, melancholy, hostility, vigor, fatigue, and confusion derived from mood assessment. These variables can negatively affect the health and training of athletes [25].

Although differences were identified between the Premier and A categories in terms of performance and psychological profiles, these findings point to opportunities to improve the comprehensive development of players in both categories. While Premier-category players have demonstrated high performance in decision-making and physical performance, they have shown deficiencies in their psychological profiles. This highlights the need for a comprehensive approach that addresses both mental and emotional well-being as well as physical performance. Decision-making ability can have a significant effect on performance at both the individual and collective levels. This suggests the importance of short-, medium-, and long-term sports training processes [57] and, in turn, invites us to consider how coaches transfer their knowledge [58,59] to sport practices to stimulate the learning and training of players.

### Limitations and Future Perspectives

One of the main limitations of this study is the small number of participants and the cross-sectional design used, precluding inferences about causality. Likewise, this prevents us from generalizing the results obtained to the rest of the athletes. Given this difficulty, it is recommended that the study sample be extended and that the study and data collection time be extended to a greater number of seasons so that data can be compared across years and categories.

By not following players over long periods, this research cannot fully capture changes in performance over time. Therefore, conducting longitudinal research in different contexts and age groups is suggested.

It is necessary to continue developing this type of research to understand how young players manifest their performance in response to physical, tactical, emotional, and psychological variables. Thus, the findings of this study could serve as input for considering diagnostic and follow-up assessments in soccer training stages and, at the same time, be relevant factors in the selection and development of sports potential. Knowing the performance profiles of athletes according to different emotional, tactical, or psychological variables is vital for the coaching staff, as it allows them to identify the weaknesses and strengths of the players according to their values. In this way, training sessions can focus on motivational levels and tactical knowledge. Moreover, the results invite us to strengthen long-term support, stimulation, and sports education strategies.

## 5. Conclusions

The differences among the physical characteristics, tactical knowledge, emotional intelligence, and mood states of youth soccer players at different competitive levels were analyzed. Multivariate analyses revealed eight groups of athletes with defined performance profiles on the basis of their playing position. The physical variables differed little between categories, whereas decision-making, game performance, melancholy, vigor, confusion, and emotional intelligence were the distinguishing factors at the player level.

This study focused on integrating physical, tactical, and psychological variables to comprehensively understand the profiles of young footballers, providing a solid foundation for developing individual and collective sports training processes focused on the health and integrity of their participants.

## Figures and Tables

**Table 1:** Activation protocol for the game performance assessment instrument.

Moment *	Distribution	Exercise Time	Interval Time
1	3 vs. 3 free play	1′30″	1′30″
2	3 vs. 3 without goalkeepers and small goals	1′30″	1′30″
3	3 vs. 3 with goalkeepers and small goals	1′30″	1′30″
4	3 vs. 3 with goalkeepers and small goals and change in direction	1′30″	1′30″

Note. * These 4 moments were established at the discretion of the research group for the assessment with the GPAI.

**Table 2:** Activation protocol for the game performance assessment instrument.

Moment *	Distribution	Group	Exercise Time	Interval Time
Moment 1	3 vs. 3 free play	3 vs. 3 Group 1	1′30″	1′30″
		3 vs. 3 Group 2	1′30″	1′30″
		3 vs. 3 Group 3	1′30″	1′30″
		3 vs. 3 Group 4	1′30″	1′30″
		3 vs. 3 Group 5	1′30″	1′30″
		3 vs. 3 Group 6	1′30″	1′30″
		Rest 5 min		
Moment 2	3 vs. 3 without goalkeepers and small goals	3 vs. 3 Group 1	1′30″	1′30″
		3 vs. 3 Group 2	1′30″	1′30″
		3 vs. 3 Group 3	1′30″	1′30″
		3 vs. 3 Group 4	1′30″	1′30″
		3 vs. 3 Group 5	1′30″	1′30″
		3 vs. 3 Group 6	1′30″	1′30″
		Rest 5 min		
Moment 3	3 vs. 3 with goalkeepers and small goals	3 vs. 3 Group 1	1′30″	1′30″
		3 vs. 3 Group 2	1′30″	1′30″
		3 vs. 3 Group 3	1′30″	1′30″
		3 vs. 3 Group 4	1′30″	1′30″
		3 vs. 3 Group 5	1′30″	1′30″
		3 vs. 3 Group 6	1′30″	1′30″
		Rest 5 min		
Moment 4	3 vs. 3 with goalkeepers and small goals and change in direction	3 vs. 3 Group 1	1′30″	1′30″
		3 vs. 3 Group 2	1′30″	1′30″
		3 vs. 3 Group 3	1′30″	1′30″
		3 vs. 3 Group 4	1′30″	1′30″
		3 vs. 3 Group 5	1′30″	1′30″
		3 vs. 3 Group 6	1′30″	1′30″
4 moments per training session	4 game situations	6 working groups	36 min of work 15 min rest between game situations	36 min

Note. * These 4 moments were assessed during four training sessions by the research group via the GPAI.

**Table 3:** Values obtained in the YYIE1 and RASTs (premier category and category A).

Variable	Category	Average—SD	*p* Value	ES
Velocity (km/h)	A	11.45 ± 0.84	0.01 **	0.40
	Premier	12.29 ± 0.74		
Distance (m)	A	1380.0 ± 720.1	0.01 **	−0.91
	Premier	2097.1 ± 677.1		
VO_2max_ (mL/min/kg)	A	47.99 ± 6.05	0.01 **	−0.91
	Premier	54.01 ± 5.68		
Time 2 (RAST)	A	6.27 ± 0.44	0.001 ***	−1.07
	Premier	6.67 ± 0.37		
Power 2 (RAST)	A	235.54 ± 66.29	0.01 **	0.39
	Premier	191.07 ± 58.59		
Time 4 (Test RAST)	A	6.66 ± 0.49	0.001 ***	0.45
	Premier	7.08 ± 0.50		
Power 4 (RAST)	A	198.52 ± 62.82	0.01 **	0.83
	Premier	160.87 ± 48.10		
Maximum power	A	264.57 ± 78.09	0.28	0.14
	Premier	249.41 ± 91.01		
Minimum power	A	165.85 ± 52.53	0.17	0.11
	Premier	152.73 ± 43.01		
Fatigue index (%)	A	37.01 ± 8.52	0.80	0.09
	Premier	37.03 ± 10.74		

Note. km/h: kilometers per hour; m: meters; mL: milliliters; min: minute; RAST: running-based anaerobic sprint test; ** *p* < 0.01; *** *p* < 0.001; ES: effect size; VO_2max_ (mL/min/kg); estimation of maximum oxygen consumption.

**Table 4:** Values obtained for the hamstring strength, COD-Timer, and speed tests.

Variable	Category	Average—SD	*p* Value	ES
Torque (Nm)	A	202.25 ± 58.42	0.71	N/A
	Premier	208.87 ± 47.72		
Angle of rupture (°)	A	116.06 ± 10.98	0.04 *	−0.72
	Premier	123.04 ± 8.42		
5-0-5 (s)	A	2.66 ± 0.16	0.70	0.10
	Premier	2.65 ± 0.13		
Average speed—5-0-5 (km/h)	A	7.75 ± 0.42	0.20	0.13
	Premier	7.92 ± 0.35		
Contact time—5-0-5 (ms)	A	404.94 ± 83.95	0.04 *	0.69
	Premier	347.71 ± 80.15		
10 m—5-0-5 (s)	A	1.98 ± 0.12	0.02 *	0.79
	Premier	1.89 ± 0.11		
COD deficit (s)	A	0.67 ± 0.13	0.08	0.79
	Premier	0.76 ± 0.14		

Note. s: seconds; km/h: kilometers per hour; ms: milliseconds, * *p* < 0.05; SD: standard deviation; ES: effect size; N/A: not applicable.

**Table 5:** Values obtained in the GPAI test (Premier category and category A).

Variable (%)	Category	Average—SD	*p* Value	ES
Game performance	A	73.31 ± 9.06	0.001 ***	−1.19
	Premier	82.13 ± 5.25		
Support	A	66.69 ± 11.60	0.001 ***	−1.27
	Premier	79.38 ± 8.31		
Decision-making	Premier	95.40 ± 5.74	0.001 ***	0.59
	A	81.90 ± 11.71		
Skill execution	A	71.33 ± 9.39	0.937	0.15
	Premier	71.61 ± 11.28		

Note. *** *p* < 0.001; SD: standard deviation; ES: effect size.

**Table 6:** Values obtained from the TEAD-R and BarOn tests.

Test	Variable	Category	Average—SD	*p* Value	ES
TEAD-R	Tension	A	33.75 ± 5.00	0.09	0.06
		Premier	45.79 ± 19.24		
	Melancholy	A	44.38 ± 8.82	0.001 ***	0.59
		Premier	64.21 ± 17.10		
	Hostility	A	40 ± 13.61	0.20	0.10
		Premier	47.37 ± 19.10		
	Vigor	A	81.88 ± 15.59	0.02 *	0.38
		Premier	92.11 ± 11.34		
	Fatigue	A	58.75 ± 18.57	0.49	0.07
		Premier	62.63 ± 14.47		
	Confusion	A	39.38 ± 10.63	0.001 ***	0.56
		Premier	61.58 ± 22.43		
BarOn	Emotional intelligence	A	75.44 ± 9.48	0.04 *	0.77
		Premier	79.53 ± 6.79		

Note. * *p* < 0.05, *** *p* < 0.001, SD: standard deviation; ES: effect size.

**Table 7:** Relationships between sets of variables (RV coefficient).

	Asym	EI	5-0-5	Cat	MS	DM	Sup	HS	GP	CMJ	SJ	RAST	YYIE1	Speed
**Asym**	1	0.11	0.10	0.07	0.11	0.18	0.25	0.07	0.13	0.07	0.07	0.06	0.10	0.05
**EI**		1	0.01	0.06	0.04	0.14	0.04	0.14	0.16	0.08	0.09	0.04	0.10	0.00
**5-0-5**			1	0.12	0.06	0.13	0.14	0.05	0.10	0.22	0.22	0.26	0.08	0.38 *
**Cat**				1	0.29	0.34 *	0.10	0.07	0.28	0.03	0.03	0.09	0.18	0.04
**MS**					1	0.07	0.23	0.03	0.04	0.03	0.05	0.04	0.01	0.07
**DM**						1	0.11	0.05	0.88 *	0.08	0.07	0.04	0.25 *	0.03
**Sup**							1	0.05	0.04	0.08	0.11	0.10	0.12	0.05
**HS**								1	0.05	0.06	0.07	0.12	0.09	0.16
**GP**									1	0.08	0.06	0.01	0.25 *	0.02
**CMJ**										1	0.83 *	0.46 *	0.07	0.33
**SJ**											1	0.44 *	0.07	0.32 *
**RAST**												1	0.08	0.48 *
**YYIE1**													1	0.04
**Speed**														1

Note. Asym (asymmetry), EI (emotional intelligence), 5-0-5 (COD-Timer 5-0-5), Cat (category), MS (mood state), DM (decision-making), Sup (support), HS (hamstring strength), CMJ (countermovement jump), SJ (squat jump), RAST (running-based anaerobic sprint test), YYIE1 (yo-yo intermittent recovery test level 1), GP (game performance). * There is proximity in the groups of variables evaluated.

**Table 8:** Profiling according to the variables analyzed.

Profiling Number	Profile 1	Profile 2
First profiling	Players with a high level of power in the SJ exhibit a correlation with strength, resulting in higher levels of speed, jump height, and flight time. Additionally, they demonstrate high levels of various powers in the RAST, which are related to the characteristics of the CMJ.	Players with high levels in variables T1 and T3 of the RAST stand out, which are associated with high levels of speed at different distances (5, 10, 15, and 20 m).
Second profiling	In this group of players, those who show high levels of support in in-game performance stand out, correlated with more effective decision-making. Additionally, they exhibit a high correlation with speed, distance covered, and estimated VO_2max_ in the YYIE1, which is associated with high levels of average speed and COD_DEF in directional changes. Preferably, players from the Premier category are found in this group.	Players with high levels in the 10 m change in direction stand out, associated with high levels of speed at 10, 15, and 20 m. This group predominantly includes players from the A category.
Third profiling	This group of players registers high levels of confusion, correlated with high levels of tension, fatigue, melancholy, hostility, and vigor. In other words, within this group, the set of MS variables is fully correlated. They also show high flight times in the asymmetry test and the highest levels in COD_DEF in a change in direction. This group is mainly composed of forward with a predominance on the left leg.	Players who exhibit high levels of flight to the right according to the asymmetry test stand out, with a high correlation with asymmetric and left contact times. This group mainly includes right-footed full-backs.
Fourth profiling	Players with high torque levels are associated with high levels in the angle of break according to the hamstring strength test. They also show high levels in left flight time, left contact time, and right flight time according to the asymmetry test. They are also players who have the highest levels in the BarOn test, with central midfielders and central defenders standing out.	Players who have higher levels of flight time in the CMJ, with a high correlation with speed and jump height. These players are associated with high levels of flight time, speed, and jump height in the SJ. This group mainly includes players in the full-back position.
Fifth profiling	In this group of players, there are those with high levels of melancholy who are also associated with high levels of hostility, confusion, and tension. These players correlate with right contact time in the asymmetry test, with central defenders standing out.	Players who have high levels of break angle in the hamstring strength are associated with high levels of time and COD_DEF in the change in direction test. They are also players who have high levels of asymmetric flight time according to the asymmetry test. Side midfielders stand out in this group.
Sixth profiling	Within this group of players, those with high levels of push distance in the asymmetry test stand out, associated with high levels of asymmetric contact time. They also correlate with higher weight levels and longer leg measurements. These players are also associated with the longest times in the change in direction test, as well as with the highest skill execution percentages in in-game performance. Goalkeepers stand out within this group of players.	Not applicable.
Seventh profiling	Players in this group have the longest flight times to the right, also associated with higher levels of flight time to the left, according to the asymmetry test. Additionally, these players exhibit high levels of fatigue, with forward standing out.	Players have the highest levels of asymmetric flight time according to the asymmetry test, with side midfielders standing among them.
Eighth profiling	According to the asymmetry test, players who have high levels of contact time to the left are associated with high levels of contact time to the right. These players are related to high levels in the BarOn test and high levels of skill execution according to the GPAI test.	Not applicable.

## Data Availability

The raw data supporting the conclusions of this article will be made available by the authors without undue reservation.
